# Long-term post-mortem studies following neurturin gene therapy in patients with advanced Parkinson’s disease

**DOI:** 10.1093/brain/awaa020

**Published:** 2020-03-23

**Authors:** Yaping Chu, Raymond T Bartus, Fredric P Manfredsson, C Warren Olanow, Jeffrey H Kordower

**Affiliations:** a1 Department of Neurological Sciences, Rush University Medical Center, Chicago, Illinois 60612, USA; a2 RTBioconsultants, Inc, San Diego, CA 92130, USA; a3 Parkinson’s Disease Research Unit, Department of Neurobiology, Barrow Neurological Institute, Phoenix, Arizona 85013, USA; a4 Departments of Neurology and Neuroscience, Mount Sinai School of Medicine, New York, NY, USA; a5 Clintrex Inc. Sarasota, Florida, USA

**Keywords:** Parkinson’s disease, gene therapy, neurturin, neuroprotection, dopaminergic neuron

## Abstract

We performed post-mortem studies on two patients with advanced Parkinson’s disease 8 and10 years following AAV2-neurturin (CERE120) gene therapy, the longest post-mortem trophic factor gene therapy cases reported to date. CERE120 was delivered to the putamen bilaterally in one case (10 years post-surgery), and to the putamen plus the substantia nigra bilaterally in the second (8 years post-surgery). In both patients there was persistent, albeit limited, neurturin expression in the putamen covering ∼3–12% of the putamen. In the putamen, dense staining of tyrosine hydroxylase-positive fibres was observed in areas that contained detectable neurturin expression. In the substantia nigra, neurturin expression was detected in 9.8–18.95% and 22.02–39% of remaining melanin-containing neurons in the patient with putamenal and combined putamenal and nigral gene delivery, respectively. Melanized neurons displayed intense tyrosine hydroxylase and RET proto-oncogene expression in nigral neurons in the patient where CERE120 was directly delivered to the nigra. There was no difference in the degree of Lewy pathology in comparison to untreated control patients with Parkinson’s disease, and α-synuclein aggregates were detected in neurons that also stained for neurturin, RET, and tyrosine hydroxylase. These changes were not associated with antiparkinsonian benefits likely due to the limited neurturin expression. This study provides the longest term evidence of persistent transgene expression following gene delivery to the CNS and the first human results when targeting both the terminal fields in the putamen as well as the originating nigral neurons.

## Teaser

Chu *et al.* present findings from post-mortem assessment of two individuals with advanced Parkinson’s disease who received gene therapy with the trophic factor neurturin, 8 and 10 years prior to death. The cases represent the longest survival times reported to date for recipients of gene therapy for Parkinson’s disease.

## Introduction

Parkinson’s disease is characterized clinically by bradykinesia, rigidity and rest tremor, and pathologically by degeneration of the nigrostriatal system with Lewy bodies (Halliday *et al.*, 1990; McRitchie *et al.*, 1997). Pathological and imaging studies indicate that there is a loss of ∼60% of neurons in substantia nigra pars compacta (SNc) at the time of onset of the classic motor features of the disease (Kordower *et al.*, 2013). Indeed, recent pathological studies note that markers of dopamine terminal activity in the dorsal striatum have largely disappeared by 4 years from the time of diagnosis (Kordower *et al.*, 2013). Thus, protection and/or augmentation of the nigrostriatal dopaminergic system at an early time point in the disease (or potentially even in the pre-motor state) is an obvious therapeutic target. Neurotrophic factors have been shown to upregulate dopamine expression in residual dopaminergic neurons and to provide protective effects in animal models of Parkinson’s disease (Kordower *et al.*, 2000; Nasrolahi *et al.*, 2018) and theoretically could enhance neuronal survival and provide symptomatic benefits in Parkinson’s disease patients.

Neurturin (NRTN) belongs to the glial cell-line derived family of neurotrophic factors which include glial derived neurotropic factor (GDNF) and has been shown to markedly enhance dopaminergic neuronal survival and behavioural function in animal models (Widenfalk *et al.*, 1997; Horger *et al.*, 1998). Through GFRα2/RET receptors, NRTN activates the MAPK and PI-3-K signalling pathways in primary neurons (Creedon *et al.*, 1997). NRTN variants are associated with Hirschsprung disease (Ruiz-Ferrer *et al.*, 2011) indicating that NRTN plays an important role in development of the peripheral nervous system. Adeno-associated virus serotype 2 (AAV-2) vector encoding the human *NRTN* gene (CERE120) was designed to deliver the *NRTN* gene to the putamen (± the substantia nigra) to protect or restore degenerating nigrostriatal dopaminergic neurons and to potentially provide clinical benefits in Parkinson’s disease (Kordower *et al.*, 2006; Gasmi *et al.*, 2007). In rodents and non-human primates, CERE120 has been shown to protect the integrity of nigrostriatal neurons from neurotoxic insult, and to increase dopamine expression in aged monkeys (Kordower *et al.*, 2006; Gasmi *et al.*, 2007; Herzog *et al.*, 2007, 2013). An initial open-label study in patients with advanced Parkinson’s disease demonstrated that CERE120 was safe and well-tolerated, and reported some clinical benefits (Marks *et al.*, 2008). However, a double-blind study testing CERE120 delivery to the putamen bilaterally in patients with advanced Parkinson’s disease failed to show significant improvement with respect to the primary outcome measure at 12 months, as compared with a sham surgery procedure (Marks *et al.*, 2010). Our initial post-mortem studies (1.5 and 3 months post-surgery) revealed some NRTN expression that was distributed over ∼15% of the putamen by volume, minimal tyrosine hydroxylase (TH) upregulation within NRTN-expressing regions, and almost none (0 to <1%) of NRTN-positive neurons in the remaining nigral neurons (Bartus *et al.*, 2011*a*,*b*). Secondary post-mortem studies on two subjects with 4-years post-surgery demonstrated 18% of the putamen with NRTN-expression, 2% of the putamen with TH upregulation, 5% nigral melanin-laden neurons with NRTN immunostaining (Bartus *et al.*, 2015). These findings indicate that there is persistent, albeit limited expression, of gene-mediated NTRN with some evidence for amplification and persistence of biological effects for at least 4 years. However, these effects were not sufficient to provide significant clinical benefit.

Following a series of prerequisite non-clinical studies in rats and non-human primates that supported targeting the substantia nigra (Bartus *et al.*, 2011*b*; Herzog *et al.*, 2011, 2013), a second double-blind clinical trial was performed to overcome the problem of limited expression as revealed by the autopsy cases. The dose and volume of AAV2-NRTN delivered to the putamen was increased by over 3-fold to enhance NRTN expression and the volume of expression. Additionally, AAV2-NRTN was also delivered directly to the substantia nigra to help ensure the degenerating cell bodies were exposed to NRTN (Bartus *et al.*, 2013). Despite these measures, CERE120 was not superior to sham in a randomized double-blind clinical trial (Olanow *et al.*, 2015*a*). No post-mortem studies have been reported on patients from this population prior to the present study.

This report describes long-term post-mortem assessments on two subjects who participated in clinical trials testing gene delivery of NTRN; one subject was from the original double-blind trial (bilateral putamenal delivery) who survived 10 years post-surgery, and a second from the second trial (bilateral putamen plus bilateral nigra) who survived 8 years following surgery. These cases represent the longest survival times of patients receiving a trophic factor therapy who have undergone post-mortem evaluations, one of which offers the first information following dual targeting of a trophic factor to both the putamen and the SNc.

## Materials and methods

### Subjects

Six brains from four patients with Parkinson’s disease (two with CERE120 therapy noted above and two without gene therapy) and two age-matched controls were included in this study (Table 1). One subject underwent CERE120 treatment delivered into the putamen bilaterally and died 10 years post-surgery (Subject 8-155). The second subject underwent CERE120 therapy delivered bilaterally into both the putamen and substantia nigra 8 years prior to death (Subject 18-24). All Parkinson’s disease subjects were diagnosed by a movement disorder specialist prior to entering the clinical trial, and the diagnosis was confirmed at post-mortem in each case by a board-certified neuropathologist. Importantly, neither of these subjects had other significant neuropathological changes and specifically, neither had any pathological change thought to be related to the gene therapy procedure. Two age-matched Parkinson’s disease subjects with comparable Unified Parkinson’s Disease Rating Scale (UPDRS) scores who did not undergo gene therapy were selected as disease controls. Two age-matched subjects without psychiatric or neurological illnesses during life or neuropathological abnormalities at post-mortem were included as healthy controls. All studies were approved by the Human Investigation Committee at Rush University Medical Centre.

**Table 1:** Demographics

Subject ID	Study	Age at death	Age at surgery	Disease duration		Dose, total vg	Pre-surgery		24 months post-surgery
				Years at death	Years before surgery		UPDRS (OFF)	UPDRS (ON)	UPDRS (OFF)
18-155 (1102)	120-02	85	75	24	14	1.8 × 10^−11^	38.0	16.5	24.0
18-24 (903-101)	120-09(ph1)	58	50	20	12	2.4 × 10^−12^	35.5	16.0	35.0
PD control 1	Rush brain bank	66	–	26	–	Untreated	39.0	–	–
PD control 2	Rush brain bank	57	–	12	–	Untreated	35.5	–	–
Normal control 1	Rush brain bank	74	–	Unknown	–	Untreated	–	–	–
Normal control 2	Rush brain bank	61	–	Unknown	–	Untreated	–	–	–

UPDRS = Unified Parkinson’s Disease Rating Scale; vg = viral genomes.

### Tissue processing

All brains were processed as described previously (Chu *et al.*, 2006). Briefly, each brain was cut into 2-cm coronal slabs and then hemisected. The slabs were fixed in 4% paraformaldehyde for 5 days at 4°C. After 24 brain blocks were sampled from one side of the brain for pathological diagnoses, the remaining brain slabs were cryoprotected in 0.1 M phosphate-buffered saline (PBS; pH 7.4) containing 2% dimethyl sulfoxide, 10% glycerol for 48 h followed by 2% dimethyl sulfoxide, and 20% glycerol in PBS for at least 2 days prior to sectioning. The fixed slabs were then cut into 18 adjacent series of 40-μm thick sections on a freezing sliding microtome. All sections were collected and stored at −20°C in a cryoprotectant solution prior to processing.

### Immunohistochemistry

An immunoperoxidase labelling method was used to examine the expression of NRTN, a major coat protein of AAV, TH, phosphorylated α-synuclein, dopamine transporter (DAT), Human Leukocyte Antigen–DR Isotype (HLA-DR, Clone: LN3), phosphor-p44/42 MAPK, and phosphor-S6 ribosomal protein. Endogenous peroxidase was quenched by 20 min of incubation in 0.1 M sodium periodate, and background staining was blocked by 1 h of incubation in a solution containing 2% bovine serum albumin and 5% either normal rabbit, goat, or horse serum. Tissue sections were immunostained for NRTN (1:250; polyclonal, R&D Systems), a major coat protein of AAV (1:500; polyclonal, Novus Biologicals), TH (1:5000; monoclonal; ImmunoStar), DAT (monoclonal; Millipore), HLA-DR (1:500; monoclonal; ThermoFisher), phosphorylated α-synuclein (phospho S129; 1:1000; Abcam), phosphor-p44/42 MAPK (1:500, polyconal, Cell Signaling), and phosphor-S6 ribosomal protein (1:500; polyclonal, Cell Signaling) antibodies. After six washes, sections were sequentially incubated for 1 h in biotinylated Rabbit anti-goat IgG (1:200; Vector) for NRTN, goat anti-rabbit for phospho S129, AAV, phosphor-p44/42 MAPK, phosphor-S6 ribosomal protein and horse anti-mouse IgG (1:200; Vector) for TH and HLA-DR followed by Elite^®^ avidin-biotin complex (1:500; Vector) for 75 min. The immunohistochemical reaction was completed with 0.05% 3,3′-diaminobenzidine and 0.005% H_2_O_2_. Sections were mounted on gelatin-coated slides, dehydrated through graded alcohol, cleared in xylene, and coverslipped with Cytoseal™ (Richard-Allan Scientific).

### Immunohistochemical controls

Immunohistochemical control experiments included omission of the primary antibodies (which control for the specificity of the staining procedure and the secondary antibody) and replacement of the primary antibodies with an irrelevant IgG matched for protein concentration. The control sections were processed in a manner identical to that described above. A pre-adsorption control experiment for NRTN and RET antibodies was also performed. Briefly, the NRTN antibody was combined with a 5-fold amount (by weight) of a recombinant human NRTN protein (1297-NE, R&D Systems) in Tris-buffered saline and incubated overnight at 4°C. The immune complexes with the antibody and blocking protein were centrifuged at 10 000*g* for 20 min. The adsorbed protein/antibody supernatant was then used in lieu of the primary antibody. The adsorption experiment of RET antibody (AF1485; R&D system) with a recombinant RET protein (1168-CR; R&D system) was performed using the above method. These experimetns resulted in total absence of staining (Supplementary Figs 1 and 2). Additionally, the staining patterns for TH (Haber and Groenewegen, 1989), and α-synuclein (Spillantini *et al.*, 1997; Chu and Kordower, 2007) were similar to what has been reported previously. All control experiments resulted in the absence of specific staining.

### Volume estimates of putamen and SNc- and NRTN-covered areas

The Cavalieri estimator (StereoInvestigator v2018, Micro-BrightField) was used to separately estimate the volume of putamen-, SNc-, and NRTN-covered areas on immunostained sections as previously described (Chu *et al.*, 2018). We sampled a series of sections from each brain using the optical fractionator principle. The interspace between sections was ∼0.72 mm. The area of interest was outlined in cross-section using a 1.25× objective. The section thickness was empirically determined in each tissue section and average of section thickness was calculated. The estimation of the area of interest was performed by means of a 50 × 50 µm point grid with a 10× objective.

### Optical fractionator estimates of NRTN- and TH-positive nigral neurons

An optical fractionator unbiased sampling design was used to estimate the number of NRTN- and TH-positive neurons within the SNc (Gundersen and Jensen 1987; Chu *et al.*, 2018). In each subject, we evaluated the SNc that extended from the caudal level of the mammillary bodies to decussation of the superior cerebellar peduncle. Approximately seven equispaced sections along the SNc were sampled from each side of brain with a sampling fraction (ssf) of 1/0.027 and a distance between sections of ∼1.44 mm. The SNc was outlined using a 1.25× objective beginning at a random starting point (StereoInvestigator v2018 software; Micro-BrightField). Counts were made at regular predetermined intervals (*x *=* *313 μm, *y *=* *313 μm), and a counting frame (70 × 70 μm = 4900 μm^2^) was superimposed on images obtained from tissue sections. The area sampling fraction (asf) was 1/0.05. These sections were then analysed using a 60× Planapo oil immersion objective with a numerical aperture of 1.4. The section thickness was empirically determined. Briefly, as the top of the section was first brought into focus, the stage was zeroed at the *z*-axis by the software. The stage then stepped through the *z*-axis until the bottom of the section was in focus. Section thickness averaged 17.21 ± 2.3 μm in the midbrain. The disector height (counting frame thickness) was 10 μm. This method allowed for 2 μm top guard zones and at least 2 μm bottom guard zones. The thickness sampling fraction (tsf) was 1/0.58. Care was taken to ensure that the top and bottom forbidden planes were never included in the cell counting.

Neuromelanin (NM) provides an endogenous marker for dopaminergic neurons, allowing for easy assessment of co-localization with NRTN immunoreactive (ir) products. NRTN-ir/NM-laden or NM-laden only (NRTN undetectable) nigral neurons were separately counted. Using stereological principles, NRTN-ir/NM-laden or NM-laden neurons in each case were sampled using a uniform, systematic, and random design. The total numbers of NRTN-ir/NM-laden neurons, NM-laden neurons only, and NRTN-ir/NM-laden plus NM-laden neurons within the substantia nigra pars compacta were calculated separately using the following formula:

(1) n=ΣQ¯ ·1/ssf · 1/asf · 1/tsf.

where ΣQ was the total number of raw counts. The number of TH-positive neuronal number in SNc neurons was determined using the same method.

The densities of NRTN-positive, TH-positive, and NM-laden nigral neurons were separately calibrated by estimating the nigral neuronal number from optical fractionator/substantia nigra volume using Cavalieri estimator (neuronal number/mm^3^). As NM-laden neurons degenerate in Parkinson’s disease, the percentage of NRTN-positive or TH-positive neurons in total NM-laden nigral neurons was calculated and compared between CERE120-treated Parkinson’s disease patients, age-matched Parkinson’s disease patients who had not undergone gene therapy, and age-matched controls. The TH-positive fibre density was calibrated by estimated fibre length from Spaceball/putamenal volume from Cavalieri estimator (fibre density/mm^3^). To assess the accuracy of this estimate, the coefficients of error (CE) were calculated according to the procedure of Gunderson and colleagues as estimates of precision (West and Gundersen 1990; Schmitz and Hof, 2000). The CE values were 0.13 ± 0.05 (range 0.10–0.15) in the Parkinson’s disease patients and 0.10 ± 0.02 (range 0.08–0.12) in the age-matched controls.

### Optical density measurement of TH immunoreactivity in putamen

Quantification of the relative optical density of putamenal TH immunoreactivity was performed using densitometry software (ImageJ 1.63; National Institutes of Health, Bethesda, MD), as described previously (Chu *et al.*, 2018). Putamenal areas with and without TH footprints were separately outlined and measured. Optical density measurements were performed in greyscale (0 represented a maximum bright image and 255 represented a maximum dark image). For each subject, ∼10 equally spaced sections through the entire putamen were sampled and evaluated. To account for differences in background staining intensity, background optical density measurements in each section were taken from corpus callosum, which lacked a TH-positive profile. The mean of these measurements constituted the background optical density that was subtracted from the optical density of TH-immunoreactive intensity measurements to provide a final optical density value.

### RNAscope *in situ* hybridization of viral genomes

To determine if low level transgene expression is due to insufficient viral genomes we also performed *in situ* hybridization against viral genomes. Human and rat (control) tissue was analysed using a custom RNAScope™ probe designed specifically against a non-transcribed portion of the recombinant AAV genome according to the manufacturer’s instructions (Advanced Cell Diagnostics) and as outlined previously (Kanaan *et al.*, 2017). RNAScope™ was developed with 3,3′-diaminobenzidine tetrahydrochloride in rat substantia nigra and in human putamen. Because of the overlap with NM, horseradish peroxidase (HRP) with a green chromogen was used to visualize genomes in the human substantia nigra.

### Double-label immunofluorescence

A double-label immunofluorescence procedure was used to determine whether RET proto-oncogene (RET) expression was affected in dopaminergic neurons by CERE120 therapy. Midbrain sections from each brain were incubated in the first primary antibody (RET, 1:250; polyclonal; R&D Systems) overnight and the donkey anti-goat antibody coupled to DyLight™ 488 (1:200, Jackson ImmunoResearch) for 1 h. After blockade for 1 h, the sections were then incubated in the second primary antibodies (TH, 1:5000) overnight, and the horse anti-mouse antibody coupled to DyLight™ 649 (1:200, Vector) for 1 h. The sections were mounted on gelatin-coated slides, dehydrated through graded alcohol, cleared in xylene, and covered using DPX (Sigma-Aldrich).

### Measurements of RET and TH fluorescence intensity

Fluorescence intensity measurements were performed according to previously published procedures (Chu *et al.*, 2012, 2018). All immunofluorescence double-labelled images were scanned with an Olympus Confocal FluoView microscope equipped with argon, helium-neon lasers, and transparent optics. With a 20× objective and a 488 and 633 nm excitation source, images were acquired at each sampling site in the SNc and were saved to a FluoView file. Following acquisition of an image, the stage moves to the next sampling site to ensure a completely non-redundant evaluation. To maintain consistency of the scanned image for each slide, the laser intensity, confocal aperture, photomultiplier voltage, offset, electronic gain, scan speed, image size, filter, and zoom were set for the background level whereby autofluorescence was not visible with a control section. These settings were maintained throughout the entire experiment. The intensity mapping sliders ranged from 0 to 4095; 0 represented a maximum black image and 4095 a maximum bright image. The RET- and TH-positive perikarya within NM-laden neurons were identified, outlined, and measured separately. Five equispaced nigral sections were sampled and evaluated. Over 150 cells were quantified for each subject. To account for differences in background staining intensity, five background intensity measurements lacking immunofluorescent profiles were taken from each section. The mean of these five measurements constituted the background intensity that was then subtracted from the measured optical density of each individual neuron to provide a final optical density value.

#### Digital illustrations

Conventional light microscopic images were acquired using an Olympus microscope (BX61) attached to a Nikon digital camera DXM1200 and stored as .tif files. Confocal images were exported from the Olympus laser-scanning microscope with FluoView software and stored as .tif files. All figures were prepared using Photoshop^®^ 7.0 graphics software (Adobe Systems, San Jose, CA). Only minor adjustments of brightness were made.

#### Data analysis

The data of optical densities and cell counts were compared from one subject with putamenal CERE120 delivery and the other with combined putamenal and nigral CERE120 delivery, to Parkinson’s disease patients without gene delivery and two normal age-matched controls. Because of the small numbers, statistical comparison between groups was not performed and only the raw data are presented in this study.

### Data availability

The data from this study will be made available upon request.

## Results

### NRTN expression

**Figure 1:**
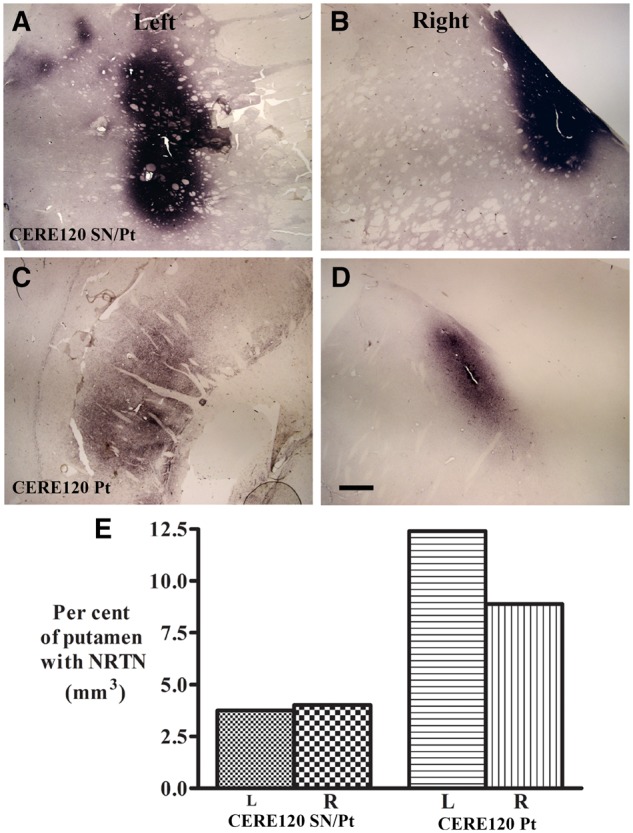
**Qualitative and quantitative evaluation for NRTN expression in putamen.** Photomicrographs of putamen from Parkinson’s disease subjects with combined nigral and putamenal CERE120 administration (CERE120 SN/Pt; **A** and **B**) or putamenal only (CERE120 Pt; **C** and **D**) illustrating neurturin (NRTN) immunohistochemical staining. Note NRTN was distributed within putamen in both cases. Case with combined putamenal and nigral CERE120 deliveries (**A** and **B**) displayed more intense in NRTN staining relative to putamenal deliveries only (**C** and **D**). Scale bar in **D **=** **1 mm (applies to all). (**E**) Histogram depicts per cent of NRTN covering left (L) and right (R) putamen.

**Figure 2:**
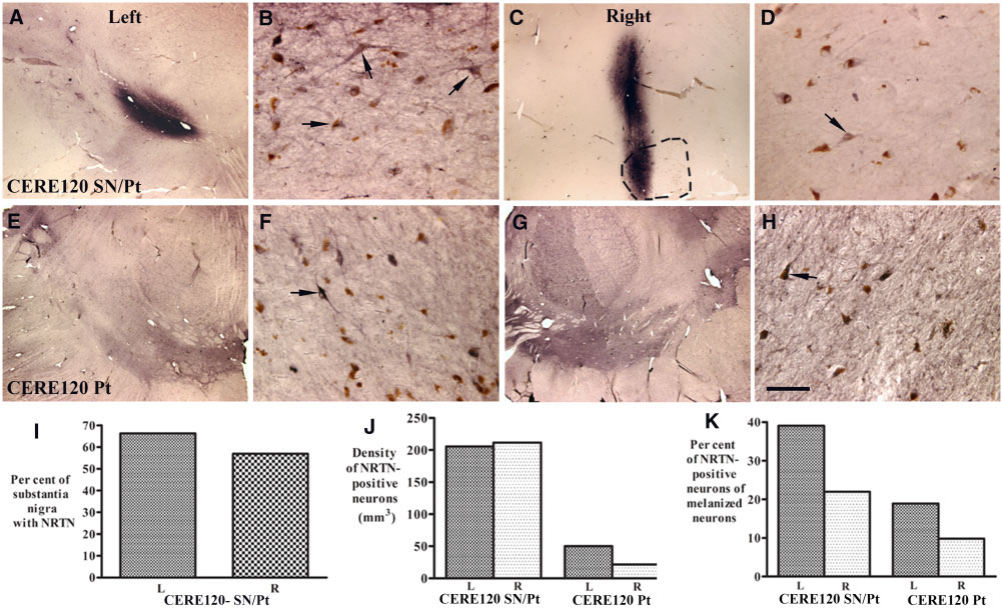
**Qualitative and quantitative evaluation for NRTN expression in substantia nigra.** Photomicrographs of substantia nigra from Parkinson’s disease subjects with combined nigral and putamenal CERE120 delivery (CERE120 SN/Pt, **A**–**D**) or putamenal delivery only (CERE120 Pt; **E**–**H**) illustrating neurturin (NRTN) immunohistochemical staining. Note NRTN were diffused into both sides of nigral region (**A** and **C**) in Parkinson’s disease with combined nigral and putamenal CERE120 delivery but not in Parkinson’s disease with putamenal delivery only (**E** and **G**). NRTN-positive neuros (arrows; **B**, **D**, **F** and **H**) were detected in substantia nigra of both Parkinson’s disease cases. Outlined area (**C**) represents substantia nigra with melanized neurons. Scale bars: **H**, **B**, **D** and **F** =** **100 µm; **A**, **C**, **E** and **G** = 1.6 mm. Cavalieri analyses demonstrated per cent of NRTN covering left (L) and right (R) substantia nigra in Parkinson’s disease with combined nigral and putamenal CERE120 delivery (**I**). (**J**) Stereological analyses revealed that the density of NRTN-positive neurons was higher in Parkinson’s disease with combined nigral and putamenal CERE120 delivery (CERE120 SN/Pt) than Parkinson’s disease with putamenal delivery only (CERE120 Pt). (**K**) There was a higher per cent of NRTN-positive neurons in remaining melanized neurons in Parkinson’s disease with combined nigral and putamenal CERE120 delivery (CERE120 SN/Pt) than Parkinson’s disease with putamenal delivery only (CERE120 Pt).

In both gene therapy-treated subjects, immunohistochemistry revealed focal regions of diffuse NRTN staining bilaterally within the putamen (Fig. 1A–D) and within the substantia nigra (Fig. 2A–H). In both locations the density of NRTN staining in the focal deposits was more pronounced in the case with CERE120 delivery to both the putamen and substantia nigra. No NRTN immunoreactivity was detected within the caudate nucleus or global pallidus in either case. In the 8-year case with combined treatment, quantification of the NRTN-stained area was estimated to cover 3.75% of the left putamen and 4.02% of the right putamen compared to 12.40% of the left putamen and 8.89% of the right putamen in the 10-year case with delivery only to the putamen (Fig. 1E). Focal deposits of NRTN staining in the substantia nigra were observed in the patient with gene delivery to this target (Fig. 2A and C) but not in the patient with gene delivery to the putamen alone (Fig. 2E and G). The NRTN-stained area was estimated to cover 66.59 (left) to 56.88 (right) per cent of the SNc (Fig. 2I).

NRTN-positive perikarya and processes were seen in both CERE120 cases (Fig. 2B, D, F and H). Quantitative stereological analyses of the substantia nigra in the patient with combined gene delivery revealed that the distribution of NRTN-positive neurons was 205.82/mm^3^ on the left and 211.77/mm^3^ on the right (Fig. 2J). In the patient with gene delivery to the putamen alone, NRTN-staining in the substantia nigra (which was dependent on retrograde transport and/or transduction from the striatum) was estimated to cover roughly 50.11/mm^3^ and 21.74/mm^3^ on the left and right sides (Fig. 2J), respectively. NTRN staining was detected in 22.02–39.13% and 9.8–18.95% of remaining nigral melanized neurons in the patients with delivery to the putamen plus substantia nigra and putamen alone, respectively (Fig. 2K). NRTN staining was not detected in the non-gene therapy-treated Parkinson’s disease controls or the age-matched healthy controls.

NRTN immunostaining was homogeneously diffuse in the putamen in both cases (Supplementary Fig. 1). There was robust NRTN staining in the nigra in the nigral inject case at an intensity that prevent localization of protein expression to a particular cell type (Fig. 2). To localize CERE120, the major coating protein (VP3) of AAV2 was examined in putamen with CERE120 delivery. Immunohistochemistry revealed that VP3 particles were mainly districted in fibres and some cells near the needle track. The fibre bundle containing VP3 vesicles extended ∼600–700 µm from needle track (Supplementary Fig. 3). The NRTN secreted from CERE120 in fibres was homogeneously diffused into putamen and substantia nigra so that NRTN-secreting cells were hardly detectable. The NRTN-immunopositive cells in substantia nigra could uptake extracellular NRTN protein although the immunohistochemical correlate of that, the halo around the positive perikarya, was never visualized.

### TH expression following CERE120 treatment in Parkinson’s disease

#### Putamen

**Figure 3:**
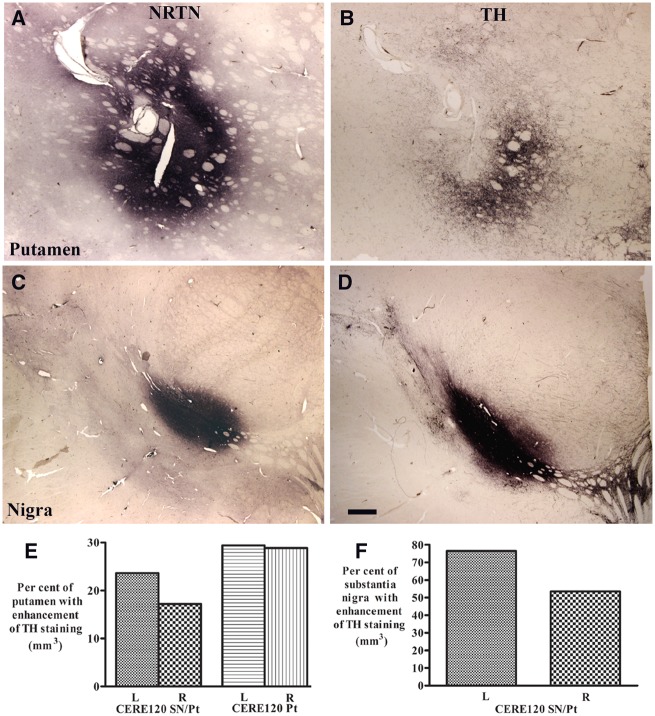
**TH expression was enhanced in area with NRTN distribution.** Photomicrographs of putamen (**A** and **B**) and substantia nigra (**C** and **D**) from Parkinson’s disease with combined nigral and putamenal CERE120 administration illustrating neurturin (NRTN; **A** and **C**) and TH (**B** and **D**) immunohistochemical staining. Note that the TH immunostaining area constituted a relatively large proportion of the NRTN-immunohistochemical footprint. Scale bar in **D **=** **1 mm (applies to all). Quantification of TH staining area revealed a proportion of putamen (**E**) and substantia nigra (**F**) displaying an enhanced TH expression.

**Figure 4:**
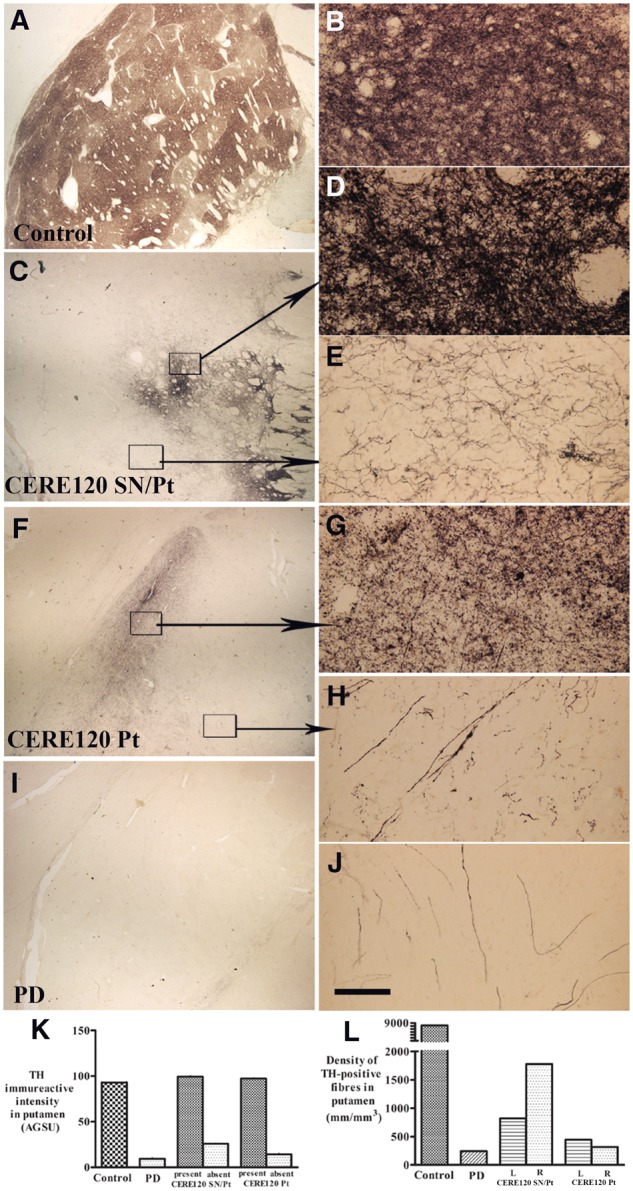
**Qualitative and quantitative evaluation for TH expression in putamen with or without CERE120 delivery.** Sections through the mid-putamen show TH-immunoreactive patterns in age-matched control (**A** and **B**), Parkinson’s disease with combined nigral and putamenal CERE120 delivery (CERE120 SN/Pt; **C**–**E**), Parkinson’s disease with putamenal delivery only (CERE120 Pt; **F**–**H**), and Parkinson’s disease without gene delivery (**I** and **J**). Note that there were dense TH-positive fibres in the CERE120 delivery areas (**C**, **D**, **F** and **G**) and scattered TH-positive fibres in the areas remote from CERE120 delivery (**E** and **H**). Scale bar in **D **=** **100 µm for **B**, **D**, **E**, **G**, **H** and **J** and 1.6 mm for **A**, **C**, **F** and **I**. (**K**) Densitometry revealed that putamenal areas with CERE120 (present) displayed higher TH-immunoreactive intensity similar to the levels of age-matched control whereas areas absent CERE120 (absent) exhibited much lower TH-immunoreactive intensity close to the levels of Parkinson’s disease without gene delivery. (**L**) Stereological analyses of TH-positive fibre density throughout putamen demonstrated that Parkinson’s disease with combined nigral and putamenal CERE120 delivery displayed higher density than the Parkinson’s disease with putamenal CERE120 delivery only. PD = Parkinson’s disease.

In CERE120-treated patients, dense TH-positive fibres, clearly above background, were observed in areas of the putamen coincident with NRTN staining (Fig. 3). Indeed, TH-staining constituted a relatively large proportion (Fig. 4C, D, F and G) of the NRTN-immunohistochemical footprint, whereas there was a comprehensive loss of TH-positive fibres (Fig. 4H) in areas without detectable NRTN staining similar to Parkinson’s disease controls (Fig. 4J), as we have previously reported (Bartus *et al.*, 2011*a*, 2015; Kordower, 2016). Quantitative densitometry confirmed that within areas of NRTN staining, TH immunoreactivity was comparable to non-Parkinson’s disease age-matched controls whereas in areas absent of NRTN expression, TH-immunoreactivity levels were similar to those seen in non-gene therapy-treated control Parkinson’s disease cases (Fig. 4K). TH immunoreactivity within the NRTN footprint was substantially greater in the case with combined delivery than in the case receiving CERE120 to the putamen alone (Fig. 4C–H). Quantitative evaluations of TH-immunoreactive fibres revealed densities ∼2–3 times greater with combined nigral and putamenal CERE120 delivery than putamen alone (Fig. 4L), which in turn was 1.3–1.8 times higher than in the Parkinson’s disease patients who did not undergo gene therapy (Fig. 4L). In comparison to age-matched healthy controls, the remaining TH-positive fibre densities were only 9.98–21.59% and 3.85–5.40% per hemisphere in the combined and putamen alone patients, respectively (Fig. 4L).

Unlike TH staining, dopamine transport (DAT) immunoreactivity was much lower in putamenal areas with NRTN expression (Supplementary Fig. 4). Moderately dense DAT-labelled fibres were observed in areas with NRTN expression and scarce in areas with absent NRTN expression in combined putamenal and nigral CERE120 delivery.

#### Substantia nigra pars compacta

**Figure 5:**
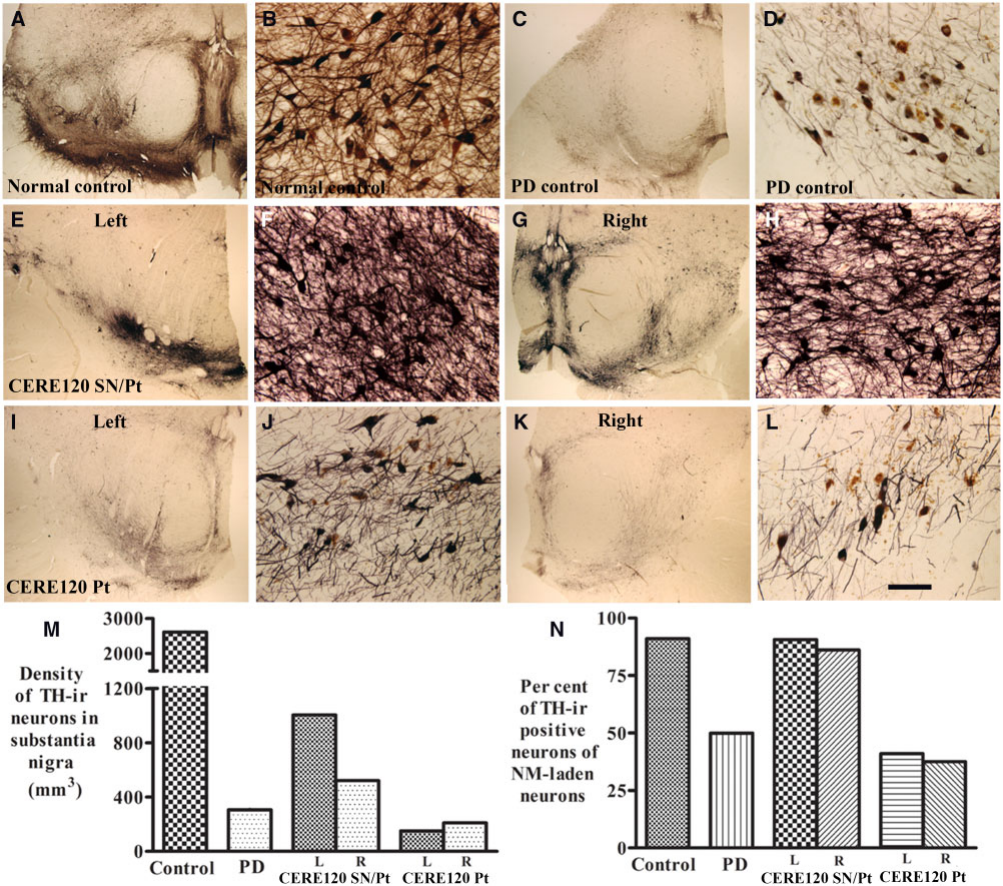
**Qualitative and quantitative evaluation for TH expression in substantia nigra with or without CERE120 delivery.** Sections from substantia nigra show TH-immunoreactive patterns in age-matched control (**A** and **B**), Parkinson’s disease without gene delivery (**C** and **D**), Parkinson’s disease with combined nigral and putamenal CERE120 delivery (CERE120 SN/Pt; left nigra: E and F; right nigra: **G** and **H**), and Parkinson’s disease with putamenal CERE120 delivery only (CERE120 Pt; left nigra: **I** and **J**; right nigra: **K** and **L**). Note that enhanced TH-staining neurons were observed in the nigral regions with CERE120 (**E**–**H**) but not in the nigral regions without CERE120 (**I**–**L**). Scale bar in **L **=** **100 µm for **B**, **D**, **F**, **H**, **G** and **J** and 1.6 mm for **A**, **C**, **E**, **G**, **I** and **K**. Stereological analyses revealed that the density of TH-positive neurons was higher in Parkinson’s disease with combined nigral and putamenal CERE120 delivery(CERE120 SN/Pt), than Parkinson’s disease with putamenal CERE120 delivery only (CERE120 Pt). (**N**) The majority of remaining melanized neurons were TH-positive in Parkinson’s disease with combined nigral and putamenal CERE120 delivery (CERE120 SN/Pt) relative to Parkinson’s disease with putamenal CERE120 delivery. PD = Parkinson’s disease; ir = immunoreactivity.

Prominent TH immunostaining was observed in the substantia nigra in the patient with combined nigral and putamenal CERE120 delivery (Fig. 5E–H). Many TH-positive neurons with abundant processes were distributed in the substantia nigra (Fig. 5E and G), In contrast, there were only a few TH-positive neurons with scarce processes and many TH-negative melanized neurons in the substantia nigra of the patient treated solely in the putamen (Fig. 5I–L). Stereological analyses revealed that densities of TH-positive neurons were 1.7–3.3 times higher with combined nigral and putamenal CERE120 delivery compared to putamen alone, which in turn was 0.49–0.68 times higher in comparison to Parkinson’s disease patients without gene delivery (Fig. 5M). A total of 86.11–90.72% of remaining melanized neurons were TH-positive following combined nigral and putamenal CERE120 delivery compared with 37.5–41.04% with putamenal CERE120 delivery (Fig. 5N).

### RET and TH expression in remaining nigral neurons with CERE120 delivery

**Figure 6:**
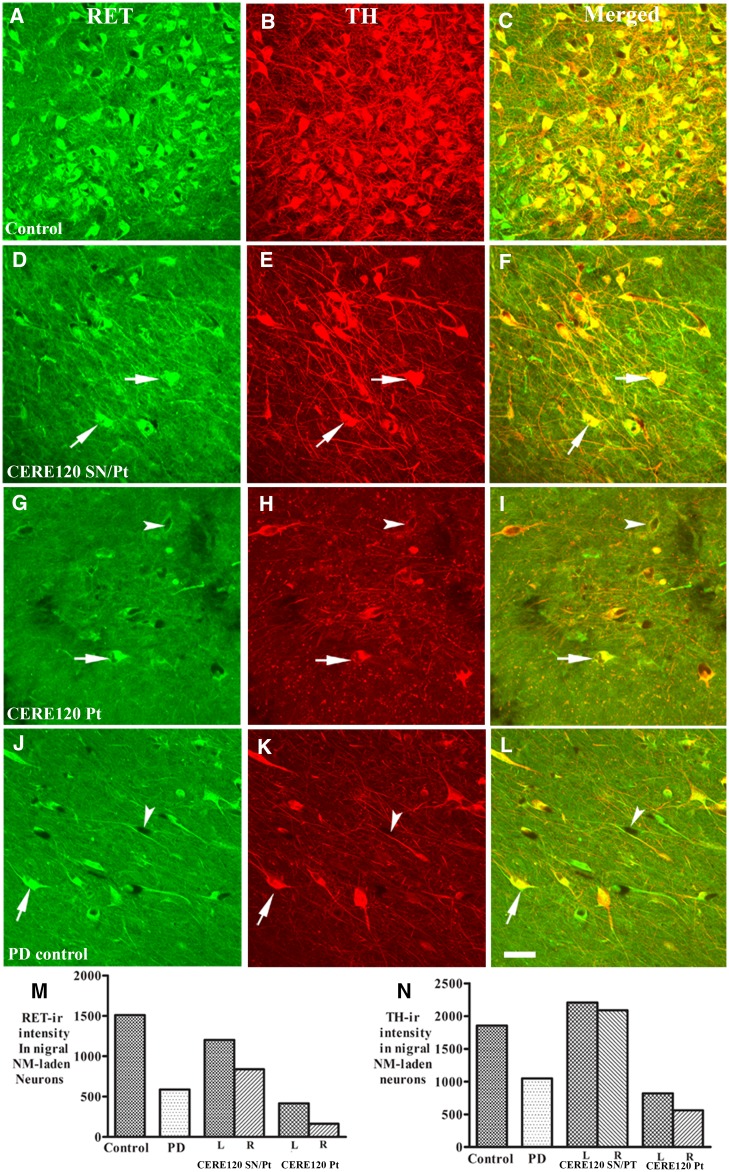
**Co-localization analyses of RET and TH expression in nigral neurons.** Confocal microscopy images of substantia nigra from age-matched control (**A**–**C**), Parkinson’s disease with combined nigral and putamenal CERE120 delivery (CERE120 SN/Pt; **D**–**F**), Parkinson’s disease with putamenal CERE120 delivery only (CERE120 Pt; **G**–**I**), and Parkinson’s disease without gene delivery (**J**–**L**) illustrating Ret (green; **A**, **D**, **G** and **J**), TH (red; **B**, **E**, **H** and **K**), and co-localization of Ret and TH (merged; **C**, **F**, **I** and **L**). In age-matched control (**A**–**C**), strong Ret (**A**) and TH (**B**) immunoreactivities were co-localized in the nigral neurons (**C**). Remaining nigral neurons in Parkinson’s disease with combined nigral and putamenal CERE120 delivery displayed intense Ret (arrows; **D**) and TH (arrows; **E**) that were co-localized in melanized neurons (arrows; **F**). However, Ret immunoreactive intensity was markedly low in subject with putamenal CERE120 delivery only (**G**) similar to Parkinson’s disease (**J**) without gene delivery. Some remaining melanized neurons had light Ret (arrows; **G**) and TH (arrow; **H**) immunostaining and other exhibited undetectable Ret (arrowheads; **G**) and TH (arrowheads; **H**). Scale bar in **L **=** **100 µm (applies to all). Quantitation of immunofluorescent intensities revealed that the optical density values of Ret (**M**) and TH (**N**) in Parkinson’s disease with combined nigral and putamenal CERE120 delivery were similar to age-matched control whereas subject with putamenal CERE120 delivery only were close to Parkinson’s disease without gene delivery. The optical densities of RET and TH immunofluorescent intensities were measured from nigral neurons with neuronal melanin. PD = Parkinson’s disease; ir = immunoreactivity.

As described above, increase of TH expression was observed in melanized nigral neurons with CERE120 delivery particularly to both the putamen and SNc. We hypothesized that maintaining TH expression is associated with the effects of the neurotrophic factor NRTN. To better understand this mechanism, we analysed the expression of the NRTN signalling receptor RET. In general, fluorescent double-labelling studies revealed that melanin-containing nigral neurons with intense RET expression displayed robust TH immunoreactivity, whereas melanized neurons with absent RET signal were TH-negative (Fig. 6). Following CERE120 delivery to both the putamen and nigra, the majority of remaining melanized neurons exhibited intense RET and TH expression approximating levels seen in age-matched controls, whereas CERE120 delivery to just the putamen showed only light RET and TH staining similar to Parkinson’s disease controls (Fig. 6A–L). Quantitative fluorescent intensities demonstrated that the levels of RET were 1.5–2.0 times higher in Parkinson’s disease with combined delivery than Parkinson’s disease controls while they were 0.27–0.71 times lower following delivery to the putamen alone (Fig. 6M). The measurement of TH immunofluorescent intensity in nigral neurons was 2.0–2.1 times higher in Parkinson’s disease with combined nigral and putamenal CERE120 delivery than Parkinson’s disease with non-gene delivery, while the levels of TH immunofluorescence intensity in Parkinson’s disease with putamenal CERE120 delivery only was similar to the Parkinson’s disease with non-gene delivery (Fig. 6N).

#### NRTN signalling pathway

NRTN signalling is mediated through a multi-component receptor system including GFRα2, RET, and glycosyl phosphatidylinositol-linked protein, that then initiates the signal pathway (Creedon *et al.*, 1997). Phospho-p-44/42 MAPK and phosphor-S6 ribosomal protein play an important role in NRTN signal pathway (Brantley *et al.*, 2008). The levels of RET expression were increased in the subject with combined CERE120 delivery relative to the subject with putamenal CERE120 delivery only. To determine whether the NRTN activates its signal pathway in this study, phospho-p-44/42 MAPK and phosphor-S6 ribosomal proteins were examined on nigral tissue from all participant subjects. Immunohistochemistry revealed more phospho-p-44/42 MAPK-positive staining around NRTN-expressing areas, but less in non-NRTN-expressing substantial nigra (Supplementary Fig. 5). Similarly, phosphor-S6 ribosomal protein-positive nigral cells were detected in Parkinson’s disease with combined CERE120 delivery but not in Parkinson’s disease with putamenal CERE120 delivery only (Supplementary Fig. 6).

### Inflammatory reaction in CERE120 delivered brains

**Figure 7:**
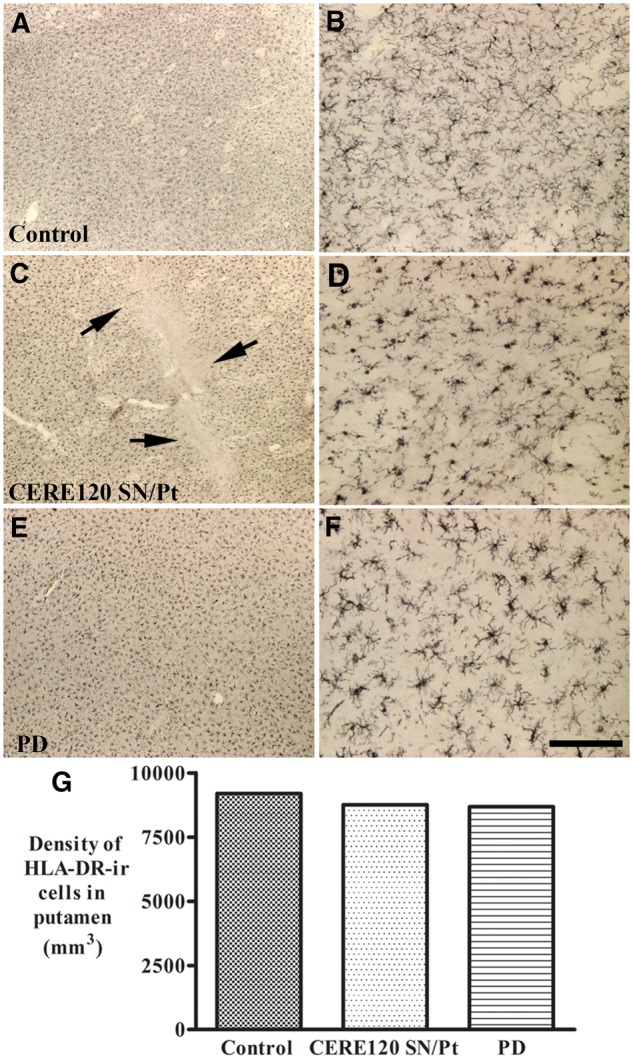
**Qualitative and quantitative evaluation for micoglial cells in putamen with or without CERE120 delivery.** Sections from putamen show human leucocyte antigen-DR isotype (HLA-DR) immunoreactive patterns in age-matched control (**A** and **B**), Parkinson’s disease with combined nigral and putamenal CERE120 delivery (**C** and **D**), and Parkinson’s disease without gene delivery (**E** and **F**). Note that the intensity and morphology of HLA-DR labelling cells in brain (**C** and **D**) with CERE120 delivery were similar to the age-matched control (**A** and **B**) and Parkinson’s disease without gene delivery (**E** and **F**). Arrows in **C** indicate a needle track for CERE120 delivery. Scale bar in **F **=** **100 µm for **B** and **D** and 1.6 mm for **A**, **C**, **E**. Stereological analyses revealed that density of HLA-DR immunoreactive (HLA-DR-ir) cells in Parkinson’s disease with CERE120 delivery was similar to age-matched control and Parkinson’s disease without gene delivery (**G**). PD = Parkinson’s disease; ir = immunoreactivity.

Previous studies have demonstrated inflammatory reactions with activated microglia associated with graft deposits following implantation of embryonic dopamine neurons into the striatum of patients with Parkinson’s disease (Olanow *et al.*, 2019). To evaluate whether there was an inflammatory reaction in CERE120 treated brains, we used HLA-DR (LN3) immunohistochemistry. While there was increased microglial staining in Parkinson’s disease controls compared to healthy controls, no additional microgliosis was observed following CERE120 delivery in the putamen (Fig. 7). In substantia nigra, the density of HLA-DR-positive cells in Parkinson’s disease with CERE120 delivery was similar to Parkinson’s disease without gene delivery. In the substantia nigra, Parkinson’s disease cases with or without CERE120 delivery displayed increased density of HLA-DR-positive cells than the age-matched controls (Supplementary Fig. 7).

### Phosphorylated α-synuclein in nigrostriatal system with CERE120 delivery

Laboratory studies have suggested that α-synuclein can downregulate RET and reduce the potential of trophic factors to provide clinical benefit (Decressac *et al.*, 2011). To assess whether pathological α-synuclein limited NRTN expression in CERE120-treated brains, putamenal and nigral tissue sections were examined using an antibody that detects 129-phosphorylated α-synuclein (P-S129-α-syn). P-S129-α-syn-positive inclusions characteristic of Lewy bodies and Lewy neurites were observed in nigral neurons and putamenal terminals, respectively (Supplementary Fig. 8), including in CERE120-delivered areas. Qualitatively, there did not appear to be differences in the number of p-S129 nigral aggregates or p-S129 putamenal fibres in relation to regions that did or did not receive CERE120 treatment or Parkinson’s disease controls. Further, α-synuclein aggregates were noted in NRTN-positive nigral neurons that contained RET and stained robustly for TH.

### Persistence of recombinant viral genomes with CERE120 delivery

Less than expected transgene expression could be the result of lower infectivity in the aged/diseased brain (Polinski *et al.*, 2016), or instability of the genome. Accordingly, to understand the extent of transduction we also evaluated CERE120 tissue for the presence of AAV genomes using RNAScope™ *in situ* hybridization revealing a non-transcribed portion of the genome. This approach allows for the detection of single genomes regardless of their transcriptional activity (Kanaan *et al.*, 2017). Surprisingly, the level of genome in both the putamen (Supplementary Fig. 9A and B) and the substantia nigra (Supplementary Fig. 9C and D) was quite low. In contrast, our standard preclinical injection paradigm in the rodent midbrain (Supplementary Fig. 9E and F) results in the detection of very high levels of AAV genomes as previously described (Benskey *et al.*, 2018).

## Discussion

Our study provides important evidence demonstrating that intrastriatal with or without intranigral gene delivery of the trophic factor NRTN (CERE120) to patients with advanced Parkinson’s disease can provide long-lasting (8–10 years) persistence of recombinant genomes with long-lasting transgene expression in putamen and substantia nigra with terminal sprouting, and focal areas of robust TH expression. This is the longest duration of transgene expression reported following trophic factor gene delivery into the CNS that has been reported to date in patients with Parkinson’s disease. The persistent transgene expression was associated with upregulation of TH and the NRTN signalling pathway in the putamenal and substantia nigral regions.

Our initial post-mortem studies on two CERE-120-treated subjects (1.5 and 3 months post-surgery) revealed ∼15% of the putamen with NRTN-expression, minimal TH upregulation, and few NRTN-positive neurons in substantia nigra (Bartus *et al.*, 2011*a*). Secondarily, post-mortem studies on two subjects at 4-years post-surgery revealed 18% of the putamen with NRTN-expression, 2% of the putamen with TH upregulation, and 5% of nigral melanin-laden neurons with NRTN immunostaining (Bartus *et al.*, 2015). In the present post-mortem evaluations, both patients (8 and 10 years post-surgery) displayed persistent NRTN expression; however, NRTN still only covered relatively small areas of the putamen (3.75 and 12.40%). In contrast, enhanced TH labelling was relatively larger (17.21–23.6% left and right hemisphere and 28.85–29.43% left and right hemisphere of putamen) and more NRTN-positive neurons were detected [9.8–18.95% putamen alone; left and right hemisphere, respectively; and 22.02–39% left and right hemisphere (putamen plus nigra)]. These studies suggest that *NRTN* gene therapy takes far longer to fulfil its promise neuroanatomically in advanced Parkinson’s disease in the concentrations of NRTN expressed than previously suspected, as a long time seems to be required for amplification with enhanced dopaminergic structures. Additionally, qualitative and quantitative studies show that these changes were much more pronounced following combined putamenal and nigral gene delivery, particularly in the substantia nigra. These data are consistent with non-clinical studies performed to support the expansion of the clinical targets (Bartus *et al.*, 2011*b*; Herzog *et al.*, 2013). Indeed, in the two hemispheres from the subject treated with both putamenal and nigral NRTN gene delivery, focal areas of the putamen with NRTN staining demonstrated intense and dense TH-positive fibre expression similar to that seen in age-matched controls, whereas putamenal areas with absent NRTN staining exhibited much lower TH-positive fibres densities similar to levels found in untreated Parkinson’s disease patients (Decressac *et al.*, 2011; Bartus *et al.*, 2015; Chu *et al.*, 2018). The subject with the higher dose of CERE120 displayed more intense NRTN and TH staining than the lower dose. However, the area of secreted NRTN from CERE120 was only diffused in a small portion of putamen in each case and the majority of putamen and nigra did not display NRTN staining. The limited diffusion of NRTN could be associated with many factors. A previous study demonstrated that all members of the GFL family (NRTN, GDNF and artemin) have high affinity for heparin sulphate proteoglycan, a transmembrane receptor in neurons especially in GABAergic neurons (Bespalov *et al.*, 2011), which likely limits NRTN diffusion. Several studies indicate that the diffusion is associated with AAV serotype (Limberis and Wilson, 2006; Kanaan *et al.*, 2017) and it is well known that the AAV2/2 capsid diffuses poorly. In fact this serotype was chosen to limit NRTN expression outside the target area. Furthermore, our results demonstrate that the major coat protein of AAV was distributed in a small area around needle track. More studies are needed to find the cause of limited NRTN diffusion. These findings must be interpreted with some caution as only one case with each treatment was available for evaluation, though the data are consistent with similar, albeit far less robust, TH expression in two prior 4-year post-dosing cases (Bartus *et al.*, 2015).

In our initial studies, CERE120 was injected bilaterally into the putamen only (Marks *et al.*, 2008, 2010). Post-mortem studies performed 1.5 months to 4 years post-surgery indicated that this approach provided only limited coverage of the putamen (Bartus *et al.*, 2011*a*, 2015). At 1.5 and 3 months we observed small areas in the putamen with intense NRTN labelling, providing only ∼15–18% coverage of the putamen by volume, but with light TH-staining fibres limited to only ∼2% coverage of the putamen by volume. At 4 years, no increase in putamenal coverage was observed, but a several-fold increase in number of TH-positive fibres was observed within NRTN-expressing areas, reflecting a mean putamenal coverage of roughly 13%, by volume (Bartus *et al.*, 2015). At the earliest time points, there were very few or no NRTN-positive nigral neurons (0 to <1% of remaining melanized neurons), and in the two 4-year cases, we saw a mean of ∼5% (range: 2–8%) of nigral neurons with detectable NRTN. From a clinical point of view, the double-blind trial failed to meet its primary end point measured at 12 months (Marks *et al.*, 2010). The autopsy data from this study suggested to us that impaired retrograde transport in these patients likely impeded a neutrophic response (Bartus *et al.*, 2011*a*). Because there are axonal transport deficits in Parkinson’s disease (Chu *et al.*, 2012) and there is a comprehensive loss of TH expression in nigrostriatal fibres by the time subjects were enrolled in the study, it was considered essential to deliver NTRN directly to nigral neurons to try to upregulate repair genes and induce a clinical benefit (Bartus, *et al.* 2013; Olanow *et al.*, 2015*b*). Unfortunately, this approach did not result in improved putamenal distribution of NRTN or TH and this double-blind study also did not meet its primary end point (change in UPDRS score during practically defined OFF periods) in comparison to a sham procedure (Olanow *et al.*, 2015*a*).

In both of the cases in the present report, putamenal areas with NRTN staining expressed extremely dense putamenal TH-positive fibres, which was enhanced within nigral neurons with addition of CERE120 delivery to the substantia nigra. However, the per cent coverage of the putamen was no greater than in previous cases even though larger gene doses and volumes were injected into the putamen in one case and into the putamen plus the nigra in the other. With combined nigral and putamenal CERE120 delivery, the density of NRTN-positive neurons in the substantia nigra was much greater (∼5.8-fold, Fig. 2J) than with putamenal delivery alone. The per cent of NRTN-positive neurons in nigral melanized neurons was also higher with combined delivery (22.02% in right nigra and 39.13% in left nigra) than with delivery to the putamen alone (9.8% in right nigra-18.95% in left nigra). Nonetheless, there was a limited level of AAV genome expression in both the putamen and the substantia nigra. Although the extent of transduction was sufficient to facilitate a high degree of focal NRTN and TH expression, the number of genomes was clearly lower than that observed in preclinical studies in which dramatic behavioural benefits were observed (Benskey *et al.*, 2018). This may be the result of a general impairment in AAV transduction in the aged brain (Polinski *et al.*, 2016), the diseased brain, and/or a lack of translation between preclinical models and Parkinson’s disease patients for CERE120 (Bartus *et al.*, 2011*b*).

Interestingly, 86.11% (right nigra) and 90.72% (left nigra) of remaining melanized neurons were TH-positive following CERE120 delivery to both substantia nigra and putamen, similar to age-matched controls (91.12%). This contrasts with delivery to putamen alone where only 37.57% (right nigra) and 41.04% (left nigra) of remaining melanized neurons displayed TH-positivity, a number similar to that seen in untreated Parkinson’s disease cases (49.92%). These observations indicate that CERE120 delivery directly to the substantia nigra can phenotypically upregulate impaired, but viable nigral neurons. However, this increase in dopaminergic markers in the area of the cell soma following nigral CERE120 delivery did not translate into a wider distribution of TH in the putamen or clinical benefits.

Under physiological conditions, NRTN preferentially binds to the GFRα2 co-receptor to assemble a complex and specific tyrosine residues of RET are phosphorylated that are brought together to initiate signal transduction and the MAP kinase signalling pathway (Creedon *et al.*, 1997; Brantley *et al.*, 2008). At the levels expressed with gene therapy, it binds to both GFRα1 and GFRα2 (Bartus *et al.*, 2015). These receptors have widespread distribution throughout the CNS and peripheral nervous system but under the conditions of the clinical trial, only the nigrostriatal system is engaged. It had previously been reported that overexpression of α-synuclein downregulates RET (Volakakis *et al.*, 2015), the NTRN receptor, and this might preclude NRTN and its homologue, GDNF, from interacting with the receptor and providing a trophic benefit. If true, this could account for the inability of our clinical trials to meet their primary endpoints. In the present study we observed large numbers of α-synuclein-positive Lewy neurites and Lewy bodies in the substantia nigra. Despite this, we observed robust RET expression and TH staining in neurons even though they contained α-synuclein-positive aggregates. These findings suggest that α-synuclein accumulation does not prevent NRTN from expressing trophic effects on nigrostriatal dopaminergic neurons, although it cannot be determined if benefits may have been attenuated. It should also be noted that Bjorklund *et al.* used gene delivery of α-synuclein in their models, which produces α-synuclein levels 8–10 times that typically seen in Parkinson’s disease patients (Decressac *et al.*, 2011). We also compared RET expression in CERE120-treated Parkinson’s disease cases, non-gene treated Parkinson’s disease cases, and age-matched non-Parkinson’s disease control brains. Double labelling studies demonstrated that TH staining was much more pronounced in nigral neurons that expressed RET whether or not they contained α-synuclein. Further qualitative and quantitative analyses revealed that RET immunoreactivity in nigral neurons in patients treated with direct nigral gene delivery were comparable to age-matched controls whereas patients treated with putamenal injections alone had RET staining similar to untreated Parkinson’s disease patients. In addition, more phosphor-p44/42 MAPK and phosphor-S6 ribosomal protein-positive neurons were only observed in direct nigral delivery of NRTN suggesting that NRTN activates its signalling pathway. These data suggest that direct nigral delivery of NTRN upregulates RET and possibly enhances dopaminergic neuronal function by regulating RET despite the presence of α-synuclein aggregates. However, it must be noted that dissociation between markers of nigrostriatal integrity and clinical benefit has been observed following a number of experimental therapies such as cell replacement and trophic factor treatment. This is true for both putative markers of dopamine upregulation as seen at post-mortem (Kordower *et al.*, 2017) and in life with fluorodopa PET (Olanow *et al.*, 2015*a*, *b*, 2019; Whone *et al.*, 2019*a*, *b*). Again, it is difficult to draw any definitive conclusions based on the small number of cases studied.

An interesting finding is the lack of a substantial inflammatory response seen in our cases while a robust inflammatory response has virtually always seen following cell replacement therapies (Kordower *et al.*, 2017; Olanow *et al.*, 2019). In the present cases, HLA-DR immunostaining showed no evidence of excess inflammation or activated microglia following gene therapy delivery to either the putamen or the substantia nigra, congruent with preclinical findings demonstrating that a single administration of AAV2 does not result in inflammation (Peden *et al.*, 2009).

In summary we demonstrate that gene delivery of NRTN can induce long-standing transgene expression in Parkinson’s disease subjects lasting for at least 8–10 years with prominent upregulation of TH in focal areas of the putamen and substantia nigra that express NRTN. Changes were more pronounced with higher gene doses injected into the putamen combined with direct nigral delivery. Importantly, no abnormal pathological or immunological changes related to the gene therapy were observed. Despite the prominent trophic effects that we observed at these time points, our patients did not experience clinical benefit in that two double-blind sham-controlled trials failed to meet their primary endpoints (Marks *et al.*, 2010; Olanow *et al.*, 2015*a*). There may be a number of explanations for this finding. First, it is likely related to the fact that gene delivery provided a mean NRTN coverage of only 18% in the prior six earlier cases and only 3.75–12.40% of the putamen in the two new cases reported here.

It is notable that in the 10-year case we injected 40 μl/putamen and in the 8-year case, 120 μl/putamen. Current AAV2-GDNF gene therapy trials are injecting close to 1000 μl per putamen using intraoperative MRI to maximize vector distribution. This technique resulted in significant increases in fluorodopa uptake on PET (Heiss *et al.*, 2019), although it remains to be established whether at the margins of the delivery there is sufficient concentrations of vector to achieve a clinical response (for more detailed discussion of this point, see Bartus and Johnson, 2017). While the clinical data are open label and thus impossible to definitively evaluate, this technique resulted in significant increases in fluorodopa uptake on PET (Heiss *et al.*, 2019), an end point not obtained in the initial phase 1 trial. It should be noted that in their PET analyses, the Heiss *et al.* study was able to define their regions of interest with the use of intraoperative gadolinium to facilitate dosing. This innovative technique was not used in our studies and with the small distribution is CERE120 seen at post-mortem we were not able to establish a positive PET signal. A second problem is the lack of residual dopaminergic fibres in patients of >5 years duration (Kordower *et al.*, 2013) and what fibres are there have impaired retrograde transport especially in cells with alpha-synuclein inclusions (Chu *et al.*, 2012). While the antibody to NTRN is relatively weak and may underestimate the extent of NRTN spread, TH staining was restricted to the areas where NRTN staining was detected; likewise, the presence of recombinant AAV genomes and the major coat protein of AAV2 was limited in distribution. It is likely that despite the use of higher gene doses in the putamen and direct injections of CERE120 into the substantia nigra in our second study, there was inadequate putamenal coverage to provide a clinical benefit, although the absence of viable TH-immunoreactive axons (Kordower, 2016), impaired retrograde transport mechanisms (Chu *et al.*, 2012), and age-related transgene expression deficiencies (Polinski *et al.*, 2016) also likely played major roles in the failure to provide a functional benefit. Our current study does, however, provide evidence that gene delivery of CERE120 can provide sustained NRTN expression over 8–10 years and induce sustained upregulation of TH expression in focal areas of the putamen and in substantia nigra. Further, robust NRTN, TH and RET expression was seen in melanized nigral neurons despite the presence of α-synuclein aggregates. This indicates that in Parkinson’s disease patients α-synuclein does not preclude NRTN from providing a trophic effect. It is also noteworthy that each of the patients in our studies had been diagnosed more than 5 years prior to gene delivery, a time when pathological studies have shown that there is almost no staining for dopamine markers in the dorsal striatum (Kordower *et al.*, 2013). Thus, many nigral neurons and dopamine terminals had likely already degenerated by this time point or were profoundly dysfunctional and incapable of responding to a trophic therapy. These observations raise the possibility that better clinical results might be obtained in patients treated at an earlier stage of Parkinson’s disease. Indeed, a *post hoc* analysis of the second of two double-blind controlled phase 2 trials of AAV2-NRTN, comparing the effects of NRTN in patients diagnosed ≤5 years previously, versus ≥10 years provided direct empirical support for that hypothesis (Olanow *et al.*, 2015*a*).

We have no explanation for why the low dose gene delivery to the putamen alone in the present study provided a greater degree of putamenal coverage than was obtained with combined therapy, although the higher dose case clearly demonstrated increased intensity of NRTN and TH expression and markedly enhanced expression in nigral neurons. If gene therapy is ever to be considered as a treatment for Parkinson’s disease, we will have to find a way to meaningfully increase TH-positive terminals in the striatum, as motor benefits are primarily dependent on TH expression in the putamen. Engineered AAV capsids to improve transduction and volumetric spread in nigrostriatal system can provide opportunities for gene therapy in Parkinson’s disease (Kanaan *et al.*, 2017; Raghunathan *et al.*, 2018).

Finally, it is worth considering the future role of trophic factors as a therapy for Parkinson’s disease (Olanow *et al.*, 2015*b*). All double-blind studies, including a recent double-blind GDNF trial (Whone *et al.*, 2019*b*), and its long term extension study (Whone *et al.*, 2019*a*) using GDNF infusions have failed to successfully reach their primary endpoints despite increases in fluorodopa uptake on PET (and in the latter case, despite widespread GDNF coverage of the putamen). Indeed, increases in fluorodopa PET in life and complete dopaminergic reinnervation on post-mortem assessments following foetal dopaminergic grafting is not necessarily associated with clinical benefit (Kordower *et al.*, 2017). Further, it remains unclear how trophic factors directed at the nigrostriatal dopamine system will benefit the non-dopaminergic features of Parkinson’s disease such as falling and dementia, which currently represent the most disabling features of the illness and the main reasons for nursing home placement (Goetz and Stebbins, 1993). More studies are needed to detect the effects of NRTN on non-dopaminergic system in Parkinson’s disease via neural cell adhesion molecule (Sandmark *et al.*, 2018) and syndecam 3 (Bespalov *et al.*, 2011). In addition, deep brain stimulation (DBS) and continuous intra-intestinal levodopa infusion provide immediate and far more impressive clinical benefits, and non-invasive therapies that treat or prevent motor complications and avoid the need for a surgical therapy are in late stage clinical trials.

## Funding

This work was supported by a grant from the Michael J. Fox Foundation, The Parkinson’s disease Foundation, and a department grant from Rush Neurological Sciences.

## Competing interests

The authors report no competing interests.

## Supplementary Material

awaa020_Supplementary_Figs_1-9

## Abbreviations

NM =: neuromelanin;
SNc =: substantia nigra pars compacta
