# Supplementary material for: Long-term post-mortem studies following neurturin gene therapy in patients with advanced Parkinson’s disease
Source: Brain. 2020 Mar 23;143(3):960–75. doi: 10.1093/brain/awaa020 (PMC7089653; doi:10.1093/brain/awaa020)
Supplement: awaa020_Supplementary_Figs_1-9 [file awaa020_supplementary_figs_1-9.pdf]

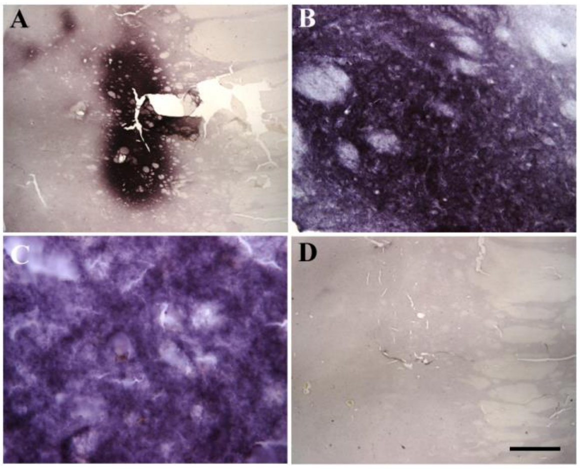

Supplementary Fig. 1. Sections showing the specificity of the NRTN antibody. Photomicrographs of sections of putamen (A) show NRTN immunoreactivity demonstrate intense. NRTN immunoreactivity (A-C). No NRTN staining was observed when the primary antibody was preadsorbed with the specific antigen (D). Scale bar in D =500µm for A, 100µm for B, and 20µm for C.

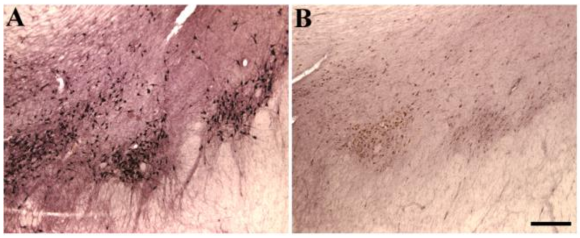

Supplementary Fig. 2. Specificity of the RET antibody. Photomicrographs of sections of substantia nigra from age-matched control illustrating RET immunoreactivity. Dark RET labelled perikarya and processes were observed in nigral neuromelanin-laden neurons (A). When the RET primary antibody was preadsorbed with the specific antigen (B, D), the neuromelanin-laden neurons displayed undetectable on RET labelling (B). Scale bar in B=400 μm for A.

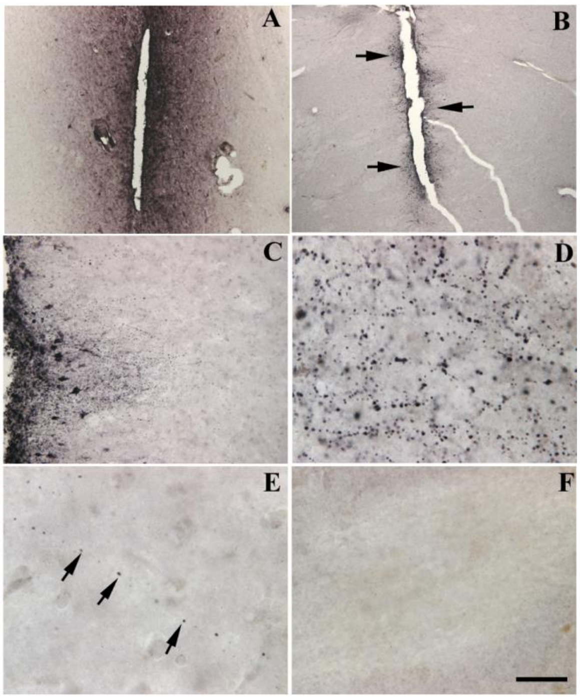

Supplementary Fig. 3. Adjacent sections from putamen with CERE120 delivery show NRTN (A) and the major major coating protein VP3 (B-F) staining. Immunohistochemistry revealed that NRTN (A) and VP3 (B) were distributed around needle track. (C) VP3 positive cells and fibers were detected near needle track (C). VP3 positive fibers were distributed about 0.6 mm from needle track (D, E). but undetectable far from needle track (over 0.8 mm; F). Arrows in B indicated needle track. Scale bar (F) = 20µm for D and E, 100µm for C, and 500µm for A and B.

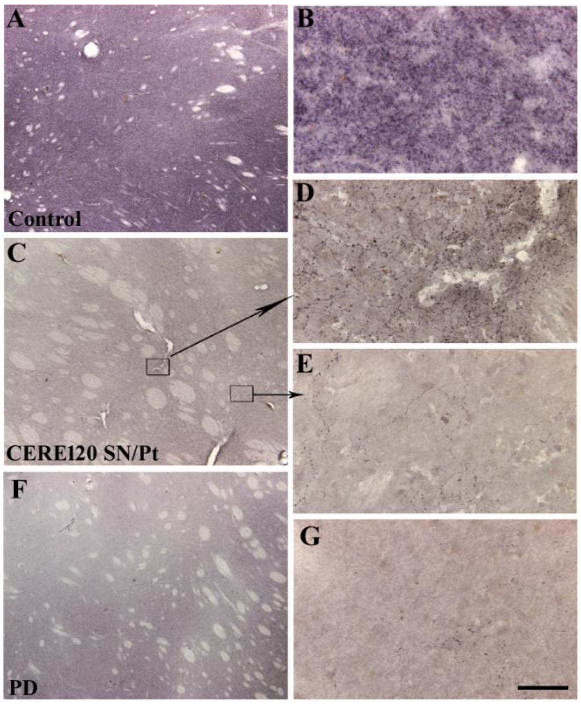

Supplementary Fig. 4. Sections from putamen show dopamine transport (DAT) immunostaining patterns in age-matched control (A, B), PD with combined nigral and putamenal CERE120 delivery (C, D, E), and PD without gene delivery (F, G). Dense DAT-labeled Fibers were homogeneously distributed to putamen in age-matched control (B). There were moderate dense of DAT-positive fibers in putamenal area with NRTN expression in a CERE-120 treated patient (D) and scarce in area with absent NRTN expression (E). Few DAT-positive fibers were observed in PD without gene delivery (G). Scale bar in (G) = 20µm for B, D, E and 200µm for A, C, F.

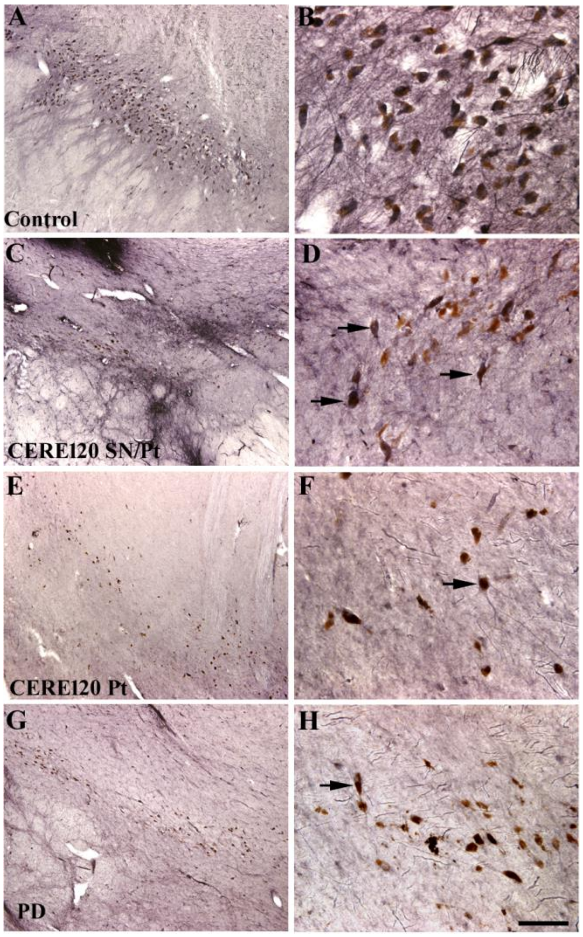

Supplementary Fig. 5. Sections from substantia nigra show robust phosphor-p44/42 MAPK immunoreactivityin age-matched control (A, B). There was expression of phosphor-p44/42 MAPK in the PD case with combined nigral and putamenal CERE120 delivery but less than control (C, D) The PD with putamenal CERE120 delivery displayed little phosphor-p44/42 MAPK (E, F) and these levels of expression were similar to a PD case without gene delivery (G, H). Scale bar (H) =100µm for B, D, F and 500µm for A, C, E, G.

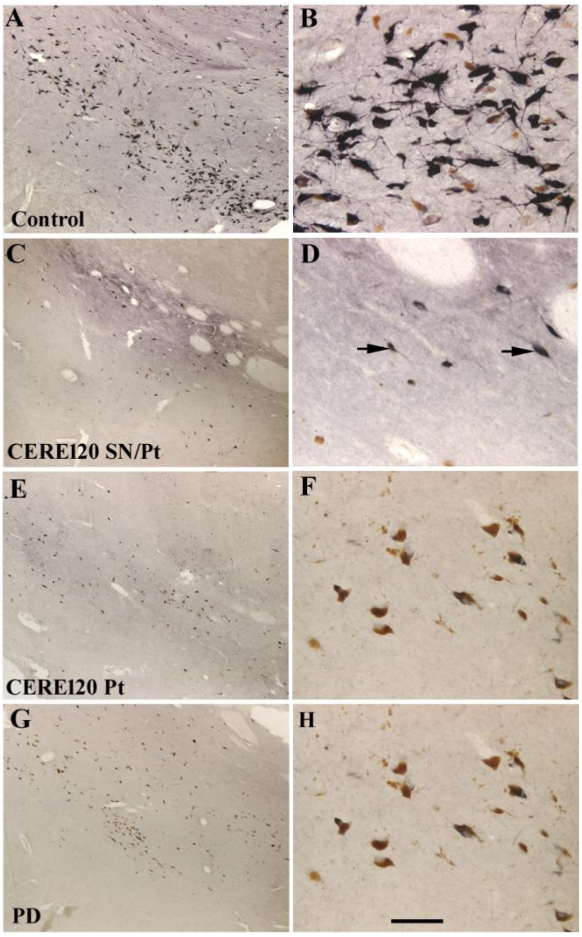

Supplementary Fig. 6. Sections from substantia nigra show robust phosphor-S6 ribosomal protein immunoreactive patterns in an age-matched control (A, B). The PD case with combined nigral and putamenal CERE120 delivery (C, D) displayed phosphor-S6 ribosomal protein staining,while the PD case with putamenal CERE120 delivery alone did not (E, F). Nor was there phosphor-S6 ribosomal proteinexpresion in a PD case without gene delivery (G, H). Scale bar (H) =100μm for B, D, F and 500μm for A, C, E, G.

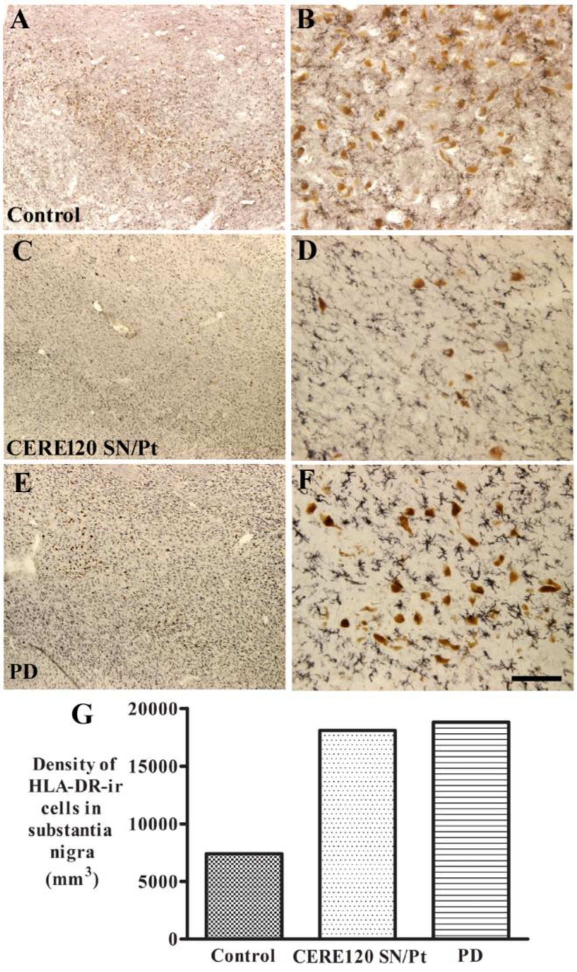

Supplementary Fig. 7. Sections from substantia nigra show human leukocyte antigen-DR isotype (HLA-DR) immunoreactive patterns in age-matched control (A, B), PD with combined nigral and putamenal CERE120 delivery (C, D), and PD without gene delivery (E, F). Note that more HLA-DR labelling cells were detected in both PD cases with (D) or without (F) CERE120 delivery relative to the age-matched control (B). Scale bar (F) =100µm for B and D and 1.6mm for A, C, E. Stereological analyses revealed that density of HLA-DR immunoreactive (HLA-DR-ir) cells in PD with CERE120 delivery was similar to PD without gene delivery but higher than age-matched control (G).

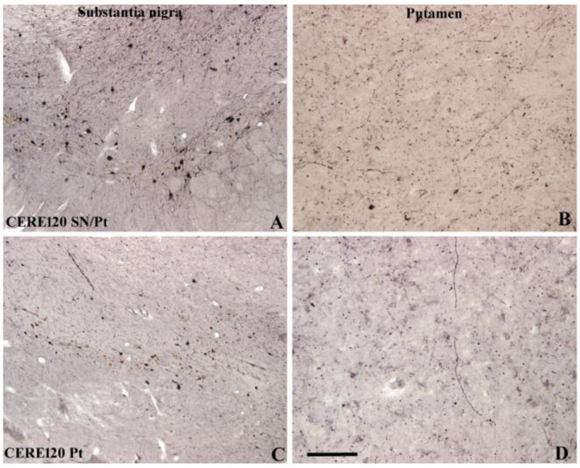

Supplementary Fig. 8. Sections from substantia nigra (A, C) and putamen (B, D) show phosphorylated a-synuclein (P-S129-αsyn) immunoreactive patterns in PD with combined nigral and putamenal CERE120 delivery (A, B) and PD with putamenal CERE120 delivery only (C, D). Note that P-S129-positive inclusions and neurites were distributed substantia nigra and putamen including NRTN covered area.

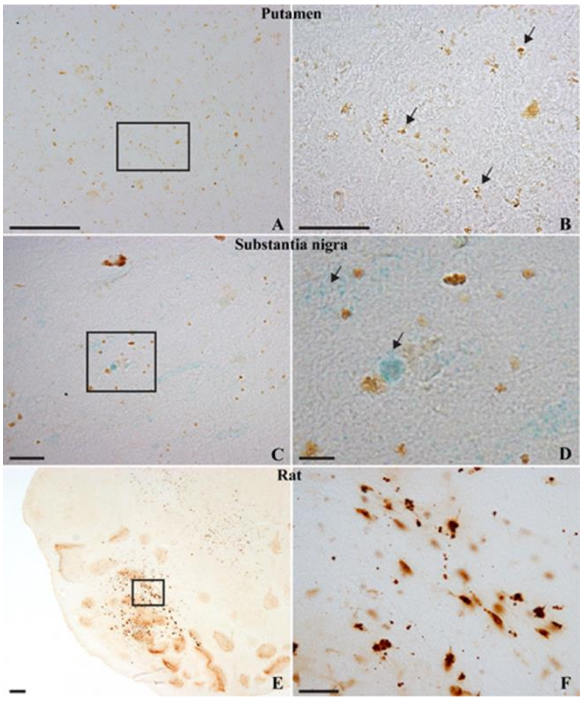

Supplementary Fig. 9. Photomicrographs of putamen (A, B) and substantia nigra (C, D) from PD with combined nigral and putamenal CERE120 administration and substantia nigra (E, F) from rat with AAV2 gene delivery showing AAV genome distribution. A. RNAscope *in situ* hybridization of AAV genome (brown) shows modest distribution of AAV genomes throughout the putamen (arrows point to examples). B. Increased magnification of area outlined in A. C. RNAscope *in situ* hybridization (developed with green pigment due to presence of neuromelanin (brown)) shows limited presence of AAV genomes in the area of the substantia nigra (arrows point to examples). D. Increased magnification of area outlined in C. E-F. Positive control AAV *in situ* hybridization from rat injected into the substantia nigra with $2x10^9$ total AAV genomes with the same probe sequence as in A-D. Scale bar (A, E) =200μm and 50μm for B-D, F).
